# Toward Empirical Evidence for Teachers’ Mental Representations of Dyadic Relationships With Students: Two Priming Experiments

**DOI:** 10.5334/pb.471

**Published:** 2019-05-09

**Authors:** Anne-Katrien Koenen, Guy Bosmans, Katja Petry, Karine Verschueren, Jantine L. Spilt

**Affiliations:** 1 School Psychology and Development in Context – KU Leuven, Leuven, BE; 1 Parenting and Special Education – KU Leuven, Leuven, BE

**Keywords:** Teacher-Student Relationships, Mental Representations, Social Information Processing, Priming

## Abstract

The attachment-based perspective on teacher-student relationships assumes that teachers internalize experiences with specific students into mental representations of dyadic relationships. Once activated, mental representations are believed to influence teachers’ affective and cognitive social information processing. Two priming experiments with 57 elementary school teachers were conducted to test these assumptions. To activate teachers’ mental representations of dyadic relationships, teachers were primed with photographs of students with whom they have a positive and negative relationship (two experimental conditions) as well as with photographs of students with whom they have a distant relationship and unknown students (two control conditions). Teachers’ responses in two different experiments –an emotion categorization task and a vignette task –were analyzed to measure differences between conditions. Mixed evidence was found for the idea that teachers’ mental representations of dyadic relationships impact their affective and cognitive information processing.

Every day, a lot of interpersonal interactions take place between a teacher and an individual student. Based on these interactions, the teacher and student are believed to develop mental representations of the relationship with the other person (Bowlby, 1969/1982; Pianta, 1999). Especially concerning conflictual teacher-student relationships, it is important to investigate teachers’ mental representations of relationships with individual students. The internalization of negative experiences into mental representations of the relationship with an individual student may activate negative affect and cognitions in teachers (including thoughts like ‘this student does it on purpose’), which may impact teacher’s sensitivity toward the student (Doumen et al., 2008; Spilt, Koomen, & Thijs, 2011). Because the idea that teachers develop mental representations of relationships with individual students has largely remained theoretical (e.g., Spilt et al., 2011), the current study aimed to provide experimental evidence for the impact of teachers’ mental representations of dyadic relationships on their emotions and cognitions.

## Attachment-Based Perspective on Teacher-Student Relationships

The idea that individuals develop mental representations of interpersonal relationships with significant others is well documented in attachment theory. Attachment theory contends that past experiences in relationships with attachment figures become internalized into mental representations of the self, the other, and the self-other relationship (Bowbly, 1969/1982; Main, Kaplan, & Cassidy, 1985). Caregivers, specifically parents as primary attachment figures of their child, develop mental representations of the caregiver-child relationship, which encompass internalized representations of the self as a caregiver, of the self in relation to the child, and of how they perceive the specific child as needing and receiving their care (Button, Pianta, & Marvin, 2001; Solomon & George, 1996). These internalized mental representations provide rules for the direction and structuring of attention and memory and, consequently, for the experiences and behavior in interaction with the attachment partner (cf., Dykas & Cassidy, 2011; Main et al., 1985). These mental representations guide the interpretations of new experiences, as well as feelings and cognitions toward the other person in future interactions (Main et al., 1985; Pianta, 1999). It is a basic quality of mental representations to automatically shape emotional, cognitive, and behavioral response patterns in concrete situations in a predictable manner (Bowbly, 1969/1982; Main et al., 1985; Pianta, 1999; Pianta, Hamre, & Stuhlman, 2003). Accordingly, caregivers’ mental representations are assumed to subsequently influence the interpretation of the child’s behavior and guide the parents’ behavior toward their child (Bretherton et al., 1989). For example, mothers’ negative mental representations of their child appear to be associated with less sensitive parenting (Button et al., 2001).

The attachment-based perspective applied to dyadic teacher-student relationships has brought to the fore the attachment functions of affective teacher-student relationships (cf., Verschueren & Koomen, 2012). Because of the physical proximity and the multiple daily interactions that teachers have with students and the affective and personal nature of these interactions, teachers are seen as professional caregivers and secondary attachment figures at school: teachers play the role of secure base and safe haven for their students at school indicating that teacher-student relationships have an attachment component (Cassidy, 2008; Verschueren & Koomen, 2012). This is assumed to be particularly the case for young students, in preschool and elementary school (Verschueren & Koomen, 2012). Professional caregivers including teachers are –like parents– believed to internalize relational experiences with children into mental representations of the relationship (Pianta, 1999; Spilt et al., 2011; Zegers et al., 2006). Teachers’ mental representations of their relationship with an individual student encompass internalized representations of the self as a teacher in various teaching roles (e.g., caregiver, instructor, disciplinarian, …), of the needs and characteristics of the student, and of the self as a teacher in relation to the student and the development of the student (Pianta, 1999; Pianta et al., 2003; Riley, 2009; Spilt & Koomen, 2009; Stuhlman & Pianta, 2002). A positive mental representation of the relationship with a student is assumed to include a more positive perception of the (needs of) student and the self in the relationship, whereas a negative mental representation is assumed to include a negative or negatively-biased set of perceptions (cf., Spilt & Koomen, 2009).

The reason of interest in teachers’ mental representations of dyadic relationships is the idea that these can explain interactions between teachers and students. The automatic activation of a particular mental representation of a teacher-student relationship in a particular situation will trigger specific feelings and cognitions (e.g., attributions) in the teacher (cf., Nummenmaa, Peets, & Salmivalli, 2008), which are assumed to guide teachers’ processing of social and situational cues. Thus, teachers’ mental representations may impact the interpretation of cues or the searching for explanations in specific situations as well as the access to possible responses and decisions how to respond in that situation (Crick & Dodge, 1994; Nummenmaa, et al., 2008). First, mental representations are assumed to impact teachers’ *affective* social information processing. Mental representations dominated by a negative connotation may induce heightened levels of negative emotional arousal (e.g., pre-activating mood-congruent cues and feelings) and may influence subsequent interactions between the persons involved (Blanchette & Richards, 2010). For example, negative affect in teachers’ representation-related narratives of dyadic relationships has been found to be related to more discipline and observable displays of negative emotions of the teacher, like anger (Pianta et al., 2003; Stuhlman & Pianta, 2002).

Second, teachers’ mental representations of dyadic teacher-student relationships may impact teachers’ *cognitive* social information processing. Mental representations dominated by a negative connotation induce negative attribution biases and negative attentional biases (Arbeau & Coplan, 2007; Blanchette & Richards, 2010; Thijs & Koomen, 2009; Sutton & Weahlty, 2003). Teachers’ negative mental representations of dyadic relationships may foster negative attributions toward the student perceiving them responsible for their misbehavior and ‘being disruptive on purpose’ (Arbeau & Coplan, 2007; Blanchette & Richards, 2010; Sutton & Weahlty, 2003; Thijs & Koomen, 2009). In addition, teachers may have more attention to and less tolerance for the non-compliant behavior of students due to the quality of the relationships: the misbehavior of students of whom they have a positive mental representation may be more readily understood and overlooked in contrast with the misbehavior of students of whom they have a negative mental representation (Arbeau & Coplan, 2007; Hamre & Pianta, 2001; Pianta, 1999).

Teachers’ mental representations of dyadic relationships may also shape teachers’ *behavioral* responses toward the specific student (Hamre & Pianta, 2001; Thijs & Koomen, 2009; Weiner, 2000). Behaviors of students of whom the teachers have negative mental representations may be more likely to be perceived negatively resulting in more attempts to control these students’ behaviors (Arbeau & Coplan, 2007; Hamre & Pianta, 2001; Ho, 2004; Stuhlman & Pianta, 2002). Indeed, teachers with a perceived conflictual relationship with their student tend to behave less warm and supportive toward this student (Roorda, Koomen, & Oort, 2012; Stuhlman & Pianta, 2002). This may, in turn, increase the negative behavior of the student, which may confirm teachers’ negative mental representations of their dyadic relationship resulting in a maladaptive pattern of transactional teacher-student interactions (Sutherland & Oswald, 2005). The impact of teachers experiencing negative teacher-student relationships with individual students can lead to a vicious circle in which negative mental representations and negative teacher-student interactions intensify each other, resulting in less sensitive behavior of the teacher and more disruptive behavior of the student (Doumen et al., 2008; Roorda et al., 2012; Sutherland & Oswald, 2005). In addition, teachers’ mental representations of dyadic relationships may trigger or temper teachers’ relational investment or motivation to spend extra time and energy in the relationship of students (Chang & Davies, 2009; Hamre & Pianta, 2001; Newberry & Davies, 2008).

Despite the assumption that teachers’ mental representations of dyadic relationships may explain teacher-student interactions, direct empirical research on the impact of teachers’ mental representations of dyadic relationships on their emotions and cognitions is scarce (Pianta et al., 2003; Spilt et al., 2011; Thijs et al., 2008). Merely indirect evidence comes from research using in-depth interviews to tap into teachers’ mental representations of dyadic relationships. For example, Stuhlman and Pianta (2002) concluded in their study using the Teacher Relationship Interview that teachers’ mental representation of the relationship with a specific student was related to their behavior toward that student. This study provided tentative cross-sectional evidence of the behavioral effects of teachers’ mental representations of their relationships with their students. The aim of the current study was to provide experimental evidence for the impact of teachers’ mental representations of dyadic relationships with individual students on teachers’ emotions and cognitions towards the specific students. One way to investigate automatic social-cognitive processes caused by internalized representations of significant relationships is by using priming techniques (Baldwin, 1994; Banse, 2001; Maier et al., 2004).

## Affective Priming of Teachers to Activate Mental Representation of Dyadic Relationships

Affective priming relies on the premise that exposure to a stimulus (prime), for which subjects possess strong affective associations, prompts the activation of the associated evaluation which impacts later judgment of emotional properties of another stimulus (target) (Fazio et al., 1986; Mograbi & Mograbi, 2012). In the current study, we primed teachers with a photograph of one of their students with whom they have a positive or negative relationship. The prime is expected to automatically activate the positive or negative content of teachers’ mental representation of dyadic relationships. The activation of this mental representation of the relationship with an individual student is thought to implicitly promote congruent affective and cognitive associations within the teacher, impacting teachers’ affect and cognitions and thus their responses to targets (Dykas & Cassidy, 2011). Thus, teachers’ mental representations of dyadic relationships can be activated using photographs of teachers’ individual students as primes to measure its impact on teachers’ affective and cognitive social information processing analyzing teachers’ evaluative responses to targets (Baldwin, 1994). The use of photographs as primes to activate mental representations of dyadic relationships has already been successfully used in several studies (e.g., Ahnert et al., 2013; Banse, 2001; Nummenmaa, et al., 2008). The method of the current study is primary based on the work of Nummenmaa and colleagues (2008) where affective priming with photographs of liked and disliked peers has been used to automatically activate adolescents’ peer-relational representations.

## The Current Study

The aim of the current experimental study was to provide evidence for the impact of teachers’ mental representations of dyadic relationships with students using affective priming. If we can find automatic responses that vary as a function of the dyadic relationship valence, this may provide evidence for the hypothesis that teachers internalize experiences with individual students into mental relationship representations which, in turn, impact their subsequent affective and cognitive responses. With the current study, we also wanted to contribute to research on teacher-student relationships that typically relies on teacher-report questionnaires of teacher-student relationships measuring explicit rather than implicit cognitions (Thijs et al., 2008). The priming method is seen as an important method in attachment research measuring implicit mental representations of attachment figures (Banse, 2001; Maier et al., 2004), but has –to our best knowledge –not been used before to test the impact of teachers’ mental representations of dyadic relationships.

In the current study, teachers were primed with photographs of their own students with whom they reported a close or conflictual relationship to activate their mental representations of dyadic relationships resulting in a positive and negative relationship condition (experimental conditions). In addition, teachers were also primed with photographs of their own students with whom they reported a distant relationship (positive nor negative valence). Teachers do not interact with each student equally frequent (positively or negatively) and for some students in the classroom, teachers will have positive nor negative feelings (Spilt & Koomen, 2009). Thus, because of the lack of proximate interactions, teachers may not develop mental representations of the dyadic relationship with students with whom they have a distant relationship (Spilt & Koomen, 2009). Consequently, no impact on teachers responses were expected for the primes with students with whom the teachers have a the distant relationship. Lastly, teachers were also primed with photographs of unknown students in order to control for the possible influence of familiarity. The distant relationship condition and the unknown condition were the two control conditions in these experiments because no activation of teachers’ mental representations of dyadic relationships was expected.

In this study in primary education, we wanted to investigate the automatic influence of teachers’ mental representations of dyadic relationship on teachers’ affective and cognitive social information processing. In the *first experiment*, we investigated the influence of teachers’ mental representations of dyadic relationships on their affective social information processing by investigating teachers’ reaction times to the target stimuli. The teachers were primed with a photograph of a student characterized with a positive (close relationship), negative (conflictual relationship), or neutral (student with distant relationship or unknown student) valence. Subsequently, teachers were asked to categorize the affective valence of the target (angry or happy facial expression of an unknown adult) as positive or negative as fast as possible. When the target was preceded by a prime of the same valence (congruent; e.g., positive prime preceded positive target), the valence of the target should be recognized faster. When the target was preceded by a prime of an opposite valence (incongruent; e.g., positive prime preceded negative target), the valence of the target should be recognized more slowly. These effects are called congruency priming effects (see more in Klauer & Musch, 2003).

Consistent with the hypothesis that teachers develop mental representations of dyadic relationships which automatically influence affective social information processing, we expected significant differences in reactions to the targets between the positive and negative teacher-student relationships on the one hand, and the two control conditions, on the other. We expected congruency priming effects (i.e., an interaction effect between condition and target): primes of positive teacher-student relationships should facilitate the evaluation of positive targets (i.e., happy facial expressions) and inhibit the evaluation of negative targets (i.e., angry facial expressions), whereas primes of negative teacher-student relationships should facilitate the evaluation of negative targets and inhibit the evaluation of positive targets compared to the two control conditions (cf., Banse, 2001; Nummenmaa et al., 2008 and see more in Klauer & Musch, 2003).

In the *second experiment*, we investigated the influence of teachers’ mental representations of dyadic relationships on teachers’ cognitive social information processing by investigating teachers’ responses to the target stimuli. After priming, teachers had to read vignettes with a description about student misbehavior where the intention of the student was ambiguous (e.g., not clear whether the student did it on purpose or not). Four questions investigated the impact of the prime on teachers’ (1) tolerance of a student’s behavior represented in the vignette, (2) attributions of low or high control to the student, (3) appraised intervention (supporting vs. setting clear limits), and (4) relational investment. We expected that teachers are more tolerant to the student’s ambiguous behavior in the vignette, attribute that the student had less control about this misbehavior, indicate more supportive strategies to intervene in the situation, and want to invest more in the relationship with the student in the positive relationship condition compared to the two control conditions. The opposite pattern was expected in the negative relationship condition (e.g., Arbeau & Coplan, 2007).

## Method

### Sample

The sample consisted of 57 teachers from elementary school classrooms (grades 1–6) in 10 elementary schools in Flanders. The majority of the teachers were female (82.46%). All teachers were born in Belgium and had obtained a Bachelor’s degree (5.26% did also obtain a Master’s degree). Teachers were on average 42 years old (range = 22–58 years; *SD* = 9.68) and had on average 19 years (range = 1–36 years; *SD* = 9.51) of teaching experience. Each teacher signed an informed consent form (the study was approved by the Ethical Board of the faculty of the authors’ university).

The experiments were conducted three weeks apart (one of the teachers quitted after the first experiment due to illness). Three teachers were excluded from the analyses because no Negative relationship condition could be identified due to (almost) no variation in Conflict on the Student Teacher Relationship Scale (see below) across their students. One teacher was partly excluded from the analyses (comparison with distant relationship control condition) because no Distant relationship condition could be identified due to (almost) no variation in Closeness on the Student Teacher Relationship Scale across their students.^1^ Accordingly, the results of the first experiment were based on 54 teachers in the Unknown control condition and 53 teachers in the Distant relationship control condition. The results of the second experiment were based on 53 teachers in the Unknown control condition and 52 teachers in the Distant relationship control condition.

The 57 teachers reported on 1238 students (51% female) between 5.72 and 13.55 years old (*M* = 8.74, *SD* = 1.73). Most of the students spoke Dutch at home (97%). The students who did not speak Dutch at home, spoke mainly French (17%), Arabic (21%) or English (10%) at home. Based on the selection criteria, 323 students (44% female) of 54 teachers between ages 6.12 and 13.55 (*M* = 8.67, *SD* = 1.70) were selected. Again, most of the students spoke Dutch at home (96%). The students who did not speak Dutch at home, spoke mainly French (33%) or English (17%) at home.

### Selecting Students Included as Primes: Conditions

For each teacher, six students of his/her classroom were selected based on teachers’ explicit reports of the quality of the teacher-student relationship using a shortened (but reliable and valid) version of the Student Teacher Relationship Scale (STRS; Koomen, Verschueren, & Pianta, 2007; Koomen et al., 2012; Pianta, 2001). Teachers scored their students on the dimensions Closeness (4 items, e.g., ‘I share an affectionate, warm relationship with this child’; α = .82) and Conflict (4 items, e.g., ‘Dealing with this child drains my energy’; α = .83) on a five-point Likert scale ranging from ‘not at all applicable’ (1) to ‘highly applicable’ (5). The STRS was completed for all students in the classroom before conducting the experiment. Students’ parents could refuse making photographs of their child with a passive informed consent form (using an opt-out procedure). Students who were not allowed to be photographed (3.86%) did not score differently from the other students on the variables Closeness (*F*(1,1236) = 1.47, *p* > .05) and Conflict (*F*(1,1236) = 1.63, *p* > .05).

For each teacher, cut-off scores based on percentiles (low: P_25_, medium: between P_25_–P_85_, and high: P_85_) of the mean scores were calculated, both for Closeness and for Conflict. The combination of the cut-off scores^2^ resulted in nine quadrants of students differing in the combination of Closeness and Conflict scores. Based on these quadrants, two students per condition were selected for each teacher (two in the Positive relationship condition, two in the Negative relationship condition, and two in the Distant relationship condition). For the ‘Positive relationship condition’, the students in the quadrant with the highest scores on Closeness (highest 15%) and the lowest scores on conflict (lowest 25%) were eligible. For the ‘Negative relationship condition’, the students in the quadrants with the highest scores on Conflict (highest 15%) and low scores on closeness (lowest 85%) were targeted. For the ‘Distant relationship condition’, students in the quadrants with low scores on both conflict (lowest 25%) and closeness (lowest 85%) were targeted. If there were not enough students identified in the quadrants using these percentiles, the students with scores nearest to these cut-off scores were also eligible (7.92%). We strived to select one boy and one girl per teacher for each condition. If more students were eligible, the highest score was preferred. If there were only students of the same sex represented in the targeted quadrants, students of the same sex were selected. This resulted in more girls (57.14%) in the Positive relationship condition and more boys (73.53%) in the Negative relationship condition, which is consistent with findings on gender differences in teacher-student relationship quality (e.g., Spilt, Koomen, & Jak, 2012).

A manipulation check on the average rating on the STRS for each condition indicated that the selection procedure was successful.^3^ The Positive relationship condition (*M_closeness_* = 4.83; *SD_closeness_* = 0.24; *M_conflict_* = 1.03; *SD_conflict_* = 0.10) and Negative relationship condition (*M_closeness_* = 3.47; *SD_closeness_* = 0.57; *M_conflict_* = 2.96; *SD_conflict_* = 0.93) differed significantly from each other on both Closeness and Conflict (paired sample t-test were all significant, *p* < .05). The Not selected students (*M_closeness_* = 4.24; *SD_closeness_* = 0.42; *M_conflict_* = 1.52; *SD_conflict_* = 0.36) differed also significantly from the students in the experimental conditions on Closeness and Conflict. The Distant relationship condition (*M_closeness_* = 3.71; *SD_closeness_* = 0.60; *M_conflict_* = 1.10; *SD_conflict_* = 0.26) scored also significantly lower on both Closeness and Conflict from the Not selected students. In addition, the Distant relationship condition did differ significantly from the Positive relationship condition on Closeness and from the Negative relationship condition on Conflict.

At least two weeks before the start of the experiments, multiple photographs of the selected students were taken at their schools. The students were asked to show neutral facial expressions. At least two independent assessors selected the most neutral photograph. The photographs were taken with a white background and at a distance of 1 meter. The photographs of the students were cropped to include only the face area of the students and resized to 6.5 mm width and 8.5 mm height (or 245 × 321 pixels) (based on Banse, 2001; Nummenmaa et al., 2008).

### Apparatus and Stimuli

The computer tasks were run on a Dell E5550 computer with a 16-inch CRT-monitor (85 Hz, resolution 1024 × 768). Affect 4 software (Hermans et al., 2005; Spruyt et al., 2010) controlled with millisecond (ms) accuracy the presentation of the stimuli and the registration of the responses and response latencies. The responses (and reaction times) were collected through clicking on mouse buttons. The primes in both experiments consisted of the photographs of the six selected students of a particular teacher (two in the Positive relationship condition, two in the Negative relationship condition, and two in the Distant relationship condition) and the photographs of two unknown same-age students (Control condition –gender balanced). The photographs of the two unknown students were selected from the Radboud Faces Database, a validated stimulus set of facial expressions (Langner et al., 2010).

### Targets

In the *first experiment*, the target stimuli consisted of 32 photographs of happy (*n* = 16) and angry (*n* = 16) facial expressions of unknown adults, also selected from the Radboud Faces Database (Langner et al., 2010; also gender balanced). In the *second experiment*, the target stimuli consisted of 24 vignettes of ambiguous misbehavior of a student to a teacher (see Appendix 1). These descriptions were based on the vignettes designed by Brophy and McCaslin (1992), confirmed to be familiar and realistic school settings in approximately 100 interviews with teachers. After translating the vignettes in Dutch, the vignettes of Brophy and McCaslin (1992) were adapted in order to make them shorter, more ambiguous (excluding references to student traits) and anonymous (not using names or references to gender). An example of such a vignette is as follows: ‘You have just presented a new lesson to the class and have assigned seat work. Everyone starts to work on the assignment. You look over the class and you see that student X is talking to the neighbor’. The vignettes were followed by four questions and each question was answered by a 10-point scale with a description of its meaning at both ends. The first question assessed a teacher’s tolerance of the student’s behavior: ‘How tolerant would you be of the student’s behavior if it were displayed in your classroom?’ (1 = ‘certainly not’/10 = ‘certainly’; Arbeau & Coplan, 2007). The second question concerned attribution of control: ‘Do you think this student has acted this way on purpose, or this student might not have meant to act this way’ (1 = ‘full control’/10 = ‘no control’; Arbeau & Coplan, 2007). The third question asks how teachers would intervene using limits setting (or support): ‘Would you intervene to make this student feel supported, or to set clear limits to the student’s behavior?’ (1 = ‘support’/10 = ‘set clear limits’; derived from Thijs, Koomen, & Van der Leij, 2006). Finally relational investment was measured: ‘How likely would you be to make an additional investment in the relationship with this student’? (1 = ‘certainly not’/10 = ‘certainly’).

### Procedure

First, at least three weeks before the first experiment, teachers completed a questionnaire with demographic questions and the STRS for each of their students in order that the six students could be identified and their photographs could be used as primes (see above). Second, the teachers completed the experiments in individual sessions in a quiet room at their school.

### First experiment

In the first experiment, teachers were told that they had to complete a computer task about the recognition of emotions. They were instructed to focus at the fixation point on the screen and were told that a sequence of two pictures at each trial would be presented. The teachers were told to ignore the first picture (the prime) and to concentrate on categorizing, as fast as possible, the facial expression of the second picture (the target) as angry or happy (cf., Nummenmaa et al., 2008). Eight practice trials preceded the experiment.

**Figure 1:**
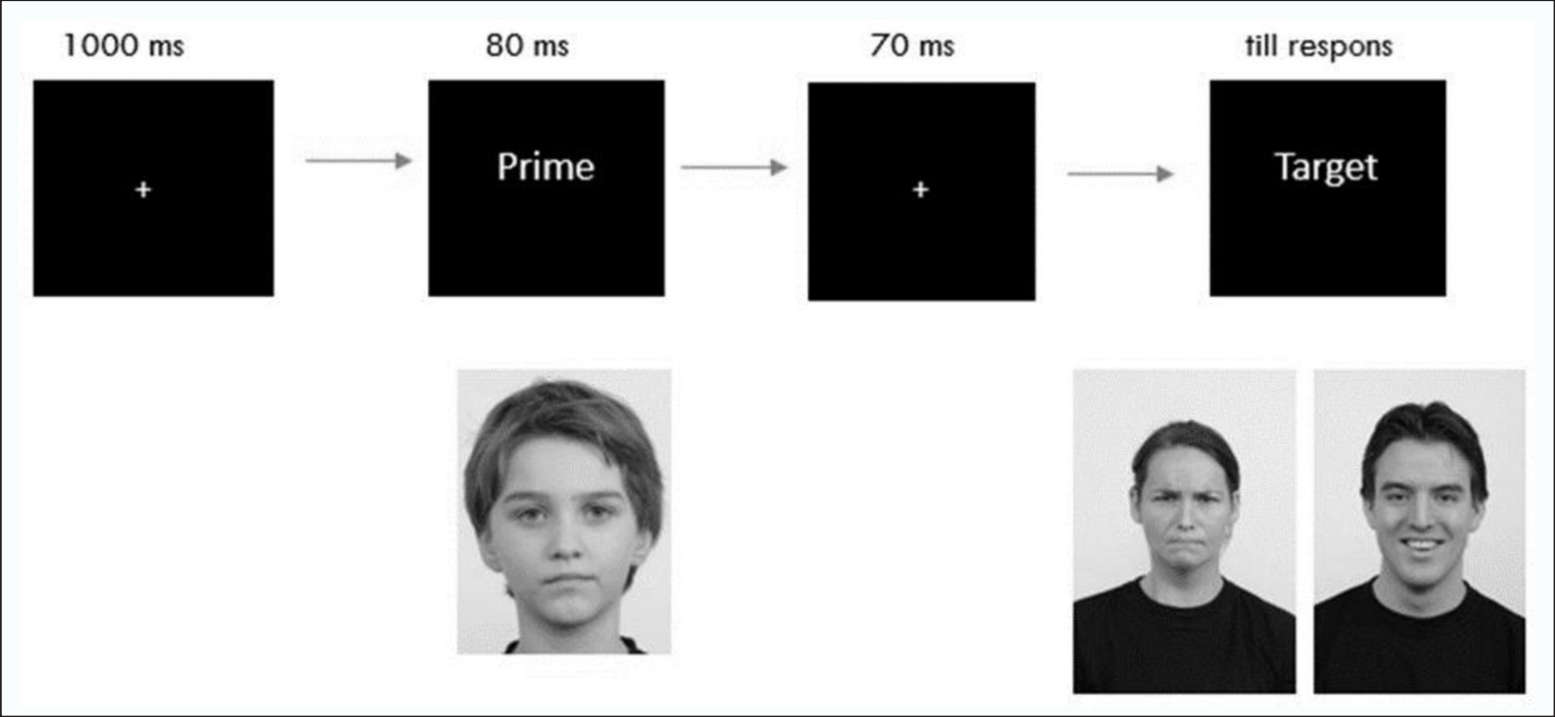
Visual representation of the time sequence of the first priming experiment.

Each trial began with a fixation point presented at the center of the screen for 1000 ms. The prime was then displayed for 80 ms, and was followed by the same fixation point displayed for 70 ms which brings the Stimulus Onset Asynchrony (SOA) on 150 ms (see Figure 1). Till now, priming research reports different conventional presentation times of prime and SOA (e.g., Andrews et al., 2011; Hermans et al., 2003; Klauer, & Musch, 2003). The presentation times of this study were chosen on the basis of Lähteenmäki and colleagues (2015) who reviewed recent critiques on priming studies and concluded that affective processing requires some level of awareness. Their study suggested that a prime of 80 ms and a SOA of 150 ms are most likely to result in reliable affective priming effects. The authors also indicated that the primes can be perceived consciously at a SOA of 150 ms, but the processing is still believed to be automatic.

After the 150 ms SOA, the target stimulus was displayed: either a happy or an angry face which disappeared when the participant responded or when 5 seconds passed by. Participants had to respond with the left (for happy targets) or right (for angry targets) mouse button with respectively their left or right index finger to categorize the target. The mouse buttons were counterbalanced over participants. Before the start of a new trial, a black screen was presented for 250 ms to reduce the retention of the target stimuli. Participants got 5 blocks of 32 targets (primes were randomly but proportionally assigned), resulting in 160 trials (with no pause).

### Second experiment

At least three weeks after the first experiment, the teachers completed the second experiment. Teachers were instructed that the computer task concerned decision making in various situations and that descriptions of behaviors of fictive students would be presented. They were instructed that they had to focus at the fixation point on the screen and that they had to carefully read the description. In line with Nummenmaa et al. (2008), the teachers were informed that they would see some pictures during the computer task, but it was stressed that the pictures were completely irrelevant to the actual experiment. Four practice trials preceded the experiment.

**Figure 2:**
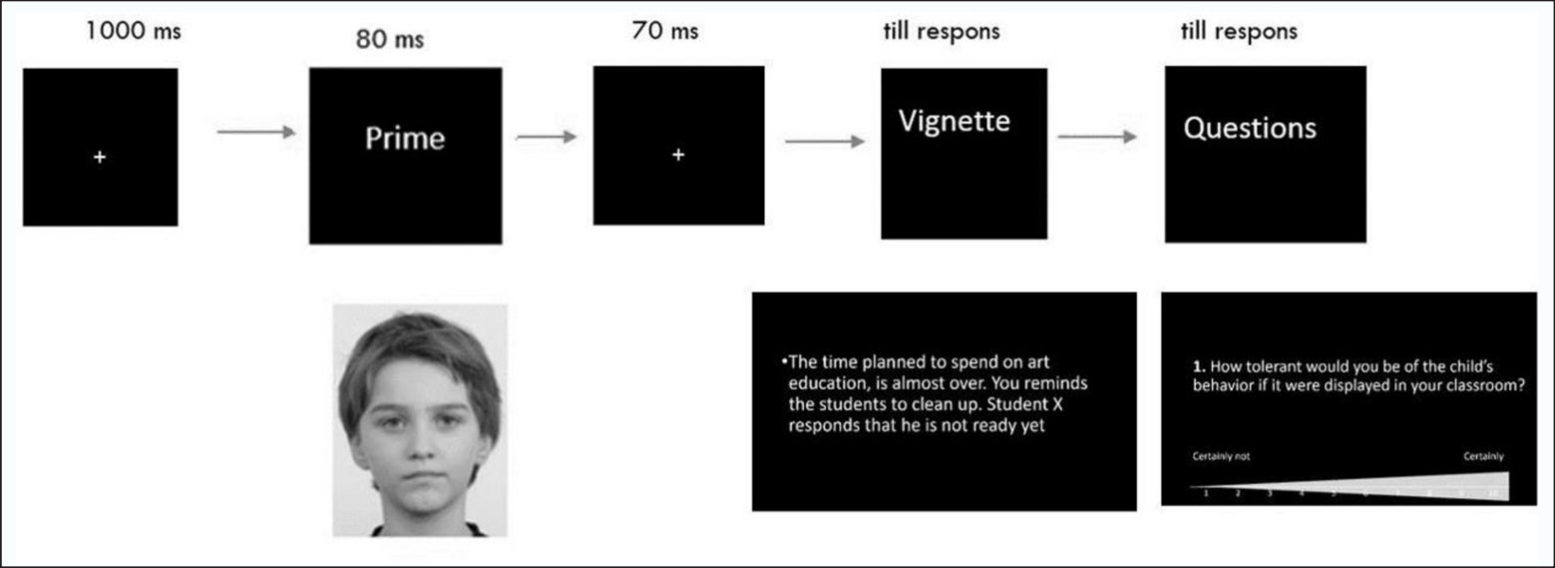
Visual representation of the time sequence of the second priming experiment.

As in Experiment 1, each trial began with a fixation point presented at the center of the screen for 1000 ms. The prime was then displayed for 80 ms, and was followed by the same fixation point displayed for 70 ms (see Figure 2). After the 150 ms SOA, the vignette was displayed. Once the participant had read the vignette, he/she continued by clicking on the ‘next’-button on the bottom of the screen (at his/her own pace). Then, the four questions were presented separately (teachers could continue by clicking on the ‘next’-button). If the participant did not answer within 150 seconds, a new trial was started. Before a new trial started, a black screen was presented for 250 ms to diminish the retention of the target stimuli. Participants got 24 vignettes (primes were randomly but proportionally assigned), each followed by the same four questions.

### Post-experimental interview

After the second experiment, the teachers were asked to answer some questions about the executed tasks, separately for Experiment 1 and Experiment 2 (e.g., “The first experiment was with the happy and angry faces, did you like that task?” but also “Which results would you expect from that task?”). Nine teachers (16%) reported some correct presumptions about at least one of the experiments in our study aiming to investigate the impact of the first pictures (primes) on their responses. However, because we assumed that the influences of the primes are automatic, we retained these teachers in the study.

## Results

### First experiment

Before the start of the analysis of the first experiment, the first block (first 32 prime-targets stimuli) was removed because pre-exposure to the stimuli is recommended to investigate affective priming effects (Calvo & Nummenma, 2007). For each teacher, the average Reaction time^4^ for each condition was calculated after the removal of outliers (> |3 *SD|*; 1.60%) and errors (not identifying the correct emotional expression; 2.12%). Table 1 depicts the descriptive statistics of teachers’ Reaction time. Two repeated measures analyses of variance (ANOVA) on Reaction time were conducted in a 2 (Target: Happy vs. Angry) × 3 (Condition: Positive vs. Negative vs. Control) within-subject design. The first analysis included Distant relationship condition as control condition and the second analysis included the Unknown condition as control condition.

**Table 1:** Descriptive Statistics of Teachers’ Responses in both Experiments.

Response	*M(SD)*	Range	1.	2.	3.

		**EXPERIMENT 1**			

0. Reaction time (in ms)	653.62(229.21)	155–2707			
		**EXPERIMENT 2**			

1. Tolerance of behavior	4.85(2.79)	1–10	–		
2. Attribution of low control	4.80(2.38)	1–10	.30*	–	
3. Limits setting (vs. support)	8.60(2.83)	1–10	–.62*	–.30*	–
4. Relational investment	6.67(2.24)	1–10	.08*	.00	–.23*

*Note:* *p < .05.

Concerning the analysis including the Distant relationship control condition, the results showed a significant main effect of Target (*F*(1,52) = 5.73, *p* = .02), indicating overall slower responses for Angry targets (*M* = 676.87; *SD* = 194.71) in comparison to Happy targets (*M* = 645.92; *SD* = 170.72). The results showed no significant main effect of the within-subject factor Condition on Reaction time (*F*(2,104) = 0.66, *p* = .52). In addition, no interaction-effect between Condition and Target was found (*F*(1.78, 92.50) = 2.20, *p* = .12 – Greenhouse-Geisser correction due to violation of sphericity with e = .89), indicating no congruency effects (i.e., the effect of condition was the same across targets). Because of the non-significant results, we decided to conduct extra within-subject contrasts in the repeated measure ANOVA to compare the Positive relationship condition and Negative relationship condition with the Distant relationship control condition (see Table 2). No significant within-subject contrasts for the Positive relationship condition (*F*(1,52) = 0.04, *p* = .84) and the Negative relationship condition (*F*(1,52) = 0.79, *p* = .38) compared to the Distant relationship control condition were found.

**Table 2:** Descriptive Statistics of Teachers’ Responses per Condition and Contrasts of the Repeated Measure ANOVA.

	Condition (valence of prime)					

	Positive relationship (P)	Negative relationship (N)	Control		Contrast compared to Control	

			Distant (D)	Unknown (U)	Distant	Unknown

	*M(SD)*	*M(SD)*	*M(SD)*	*M(SD)*		

Target						

		**EXPERIMENT 1**				

*Reaction time*						
Happy	650.51(162.99)	642.23(157.75)	645.01(161.43)	623.03(141.27)		
Angry	668.35(176.56)	686.01(201.48)	676.25(206.10)	664.80(181.05)		
Total	659.43(169.78)	664.12(179.62)	660.63(183.56)	643.92(161.16)	P = D	P > U*
					N = D	N > U*
		**EXPERIMENT 2**				

*Response*						
Tolerance of behavior	4.81(1.49)	4.58(1.23)	5.01(1.26)	4.96(1.24)	P = D	P = U
					N < D*	N < U*
Attribution of low control	4.84(1.14)	4.72(1.22)	4.83(1.38)	4.89(1.20)	P = D	P = U
					N = D	N = U
Limits setting (vs. support)	5.52(1.31)	5.76(1.29)	5.63(1.51)	5.54(1.32)	P = D	P = U
					N = D	N = U
Relational investment	6.58(1.50)	6.58(1.33)	6.63(1.52)	6.50(1.63)	P = D	P = U
					N = D	N = U

*Note:* * *p* < .05; All the within-subject contrasts were controlled for familywise error rate due to multiple comparisons using the Benjamini–Hochberg procedure and were still significant at the significance level of .05 (cf., Benjamini & Hochberg, 1995); Positive relationship condition = high on Closeness, low on Conflict; Negative relationship condition = low on Closeness, high on Conflict; Distant relationship control condition = low on Closeness, low on Conflict; Unknown control condition = unknown student.

Concerning the analysis including the Unknown control condition, the results showed a significant main effect of Target (*F*(1,53) = 8.38, *p* < .01), indicating overall slower responses for Angry targets (*M* = 673.05; *SD* = 186.36) in comparison to Happy targets (*M* = 638.59; *SD* = 154.00). The results showed also a significant main effect of the within-subject factor Condition on Reaction time (*F*(2,106) = 7.91, *p* < .01). No interaction-effect between Condition and Target was found (*F*(2,106) = 2.21, *p* = .12), indicating no congruency effects (i.e., the effect of condition was the same across targets). Because of the non-significant interaction-effect, we decided to conduct extra within-subject contrasts in the repeated measure ANOVA (see Table 2). Significant within-subject contrasts for the Positive relationship condition (*F*(1,53) = 6.86, *p* = .01; *d* = 0.09) and the Negative relationship condition (*F*(1,53) = 13.50, *p* < .01; *d* = 0.12) compared to the Unknown control condition were found. Teachers were slower in recognizing the emotional expressions in the Positive and Negative relationship conditions compared to the Unknown control condition.

### Second experiment

Table 1 depicts the descriptive statistics of teachers’ responses on the four questions in the vignette task: Tolerance of student’s behavior, Attribution of low control, Limits setting (vs. support), and Relational investment. For each teacher and each question, the average Response for each condition was calculated. Planned within-subject contrasts in repeated measures ANOVAs on teachers’ Response were conducted, for each question separately, to investigate differences between the Positive relationship and Negative relationship versus the two control conditions (Unknown and Distant relationship) (see Table 2).

Concerning the analysis including the Distant relationship control condition, the within-subject contrasts of the Positive and Negative relationship condition compared to the Distant relationship control condition revealed a significant effect for the Negative relationship condition (*F*(1,51) = 6.99, *p* = .01; *d* = 0.35), but not for the Positive relationship condition (*F*(1,51) = 0.95, *p* = .33) for the first question about Tolerance of student’s behavior. Accordingly, teachers’ responses were significantly lower on tolerance in the Negative relationship condition in comparison to Distant relationship control condition. Regarding the second question about Attribution of low control, no significant within-subject contrasts of the Positive (*F*(1,51) = 0.02, *p* = .88) and Negative (*F*(1,51) = 0.53, *p* = .47) relationship conditions compared to the Distant relationship control condition were found. Also for the third question about Limits setting (vs. support), no significant within-subject contrasts of the Positive (*F*(1,51) = 0.26, *p* = .61) and Negative (*F*(1,51) = 0.64, *p* = .43) relationship conditions compared to the Distant relationship control condition were found. Finally, for the fourth question about Relational investment, no significant within-subject contrasts of the Positive (*F*(1,51) = 0.38, *p* = .54) and Negative (*F*(1,51) = 0.54, *p* = .47) relationship conditions compared to the Distant relationship control condition were found.

Concerning the analysis including the Unknown control condition, the within-subject contrasts of the Positive and Negative relationship condition compared to the Unknown control condition revealed a significant effect for the Negative relationship condition (*F*(1,52) = 5.96, *p* = .02; *d* = 0.31), but not for the Positive relationship condition (*F*(1,52) = 0.55, *p* = .46) for Tolerance of student’s behavior. As seen in the comparison with the Distant relationship control condition, teachers’ responses were significantly lower on tolerance in the Negative relationship condition in comparison to the Unknown control condition. Regarding the second question about Attribution of low control, no significant within-subject contrasts of the Positive (*F*(1,52) = 0.10, *p* = .75) and Negative (*F*(1,52) = 0.90, *p* = .35) relationship conditions compared to the Unknown control condition were found. Also for the third question about Limits setting (vs. support), no significant within-subject contrasts of the Positive (*F*(1,52) = 0.01, *p* = .94) and Negative (*F*(1,52) = 1.16, *p* = .29) relationship conditions compared to the Unknown control condition were found. Finally, for the fourth question about Relational investment, no significant within-subject contrasts of the Positive (*F*(1,52) = 0.22, *p* = .64) and Negative (*F*(1,52) = 0.23, *p* = .64) relationship conditions compared to the Unknown control condition were found.

### Post-experimental interview

The conclusions on *p* < .05 significance level remained the same when we excluded the nine teachers who indicated some suspicion about the intention of the study from the analyses. These results without the nine suspicious teachers can be found in Appendix 3.

## Discussion

The current study introduced a new research method to investigate teachers’ mental representations of dyadic relationships. In this study, affective priming was used to test the hypothesis that teachers’ mental representations of dyadic relationships impact the affective and cognitive information processing of the teacher. Two priming experiments were conducted which yielded mixed support for this theoretical idea.

### Empirical Evidence for Teachers’ Mental Representations of Dyadic Relationships

Relying on the attachment-based perspective and consistent with research on parent-child relationships, researchers studying teacher-student relationships have suggested that teachers internalize experiences with specific students into mental representations of dyadic relationships (Pianta et al., 2003). A mental representation of a dyadic relationship is a set of internalized feelings and beliefs that a teacher has formed about his or her relationship with a student, which is automatically activated in interactions with that student. Once activated, a mental representation of a dyadic relationship is believed to influence the teacher’s social information processing and to shape the teacher’s emotional and behavioral responses (Hamre & Pianta, 2001; Pianta et al., 2003; Roorda et al., 2012; Thijs & Koomen, 2009). However, direct empirical evidence investigating the impact of teachers’ mental representations of dyadic relationships has been lacking. The current study investigated if teachers’ automatic responses varied as a function of relationship valence in two affective priming experiments.

### Impact of teachers’ mental representations of dyadic relationships on teachers’ processing of affective social information (Experiment 1)

The first priming experiment investigated the impact of teachers’ mental representations of dyadic relationships on teachers’ processing of affective social information. The (small) significant priming effects between the students with whom the teachers had a positive or negative relationship in comparison with unknown students (i.e., delayed reaction times in the positive and negative relationship conditions), regardless of their direction, may indicate that the affective meaning of the prime had activated teachers’ mental representations of the dyadic relationship with the specific student which selectively influenced the teachers’ responses to the targets (Hermans et al., 2003). However, no priming effects were found between known students: the students with whom the teachers had a positive or negative relationship in comparison with the students with whom the teachers had a distant relationship. Because these priming effects were not seen compared to the students with whom the teachers had a distant relationship and also no congruency effects were found, the first experiment could not provide evidence for the impact of teachers’ mental representations on teachers’ affective social information processing. The results of the first experiment only indicated that teachers’ affective social information processing was delayed when they were primed with students they knew, suggesting that familiarity may induce more arousal or attention, explaining the subsequent slower responses (cf., Hermans et al., 2003; Whittlesea & Williams, 1998).

The null results in the first experiment may be due to the design of the study. For example, an explanation for the slower responses in positive, negative, and distant relationship conditions may be that the responses of the teachers were delayed due to accuracy strategies. When teachers focused on the accuracy of their responses (only 2.12% of errors and also indicated by the teachers themselves during the experiment), it might have been time-consuming to disentangle the activated affective associations of a familiar prime from the target to arrive at a decision, resulting in overall slower reaction times (Hermans et al., 2003). Therefore, it would be interesting to replicate this experiment using target tasks with no need for accuracy (e.g., neutral targets, Banse 1999). As in the second experiment, a target task inducing ambiguity (introducing unclarity about the ‘correct’ answer) may also address this limitation.

### Impact of teachers’ mental representations of dyadic relationships on teachers’ processing of cognitive social information (Experiment 2)

The second priming experiment investigated the automatic effect of teachers’ mental representations of dyadic relationships on teachers’ responses to students’ misbehavior in ambiguous situations. Teachers were significantly less tolerant of misbehavior of the student in the negative relationship condition in comparison to the two control conditions. These results indicated that negative mental representations of teachers, activated by students with whom they reported a negative relationship, negatively impacted the cognitive social information processing of the teacher. Teachers may have more attention to the negative behavior of students activated by conflictual teacher-student relationships becoming less tolerant to non-compliant behavior of students (Hamre & Pianta, 2001; Pianta, 1999). In turn, less tolerance of students’ misbehavior may impact teachers’ perceptions of the needs of the student as well as teachers’ (sensitive) behavior (Hamre & Pianta, 2001; Thijs & Koomen, 2009). If teachers develop negative mental representations of dyadic relationships that negatively impact their cognitive social information processing, research and interventions have to pay attention to teachers’ mental representations to alter negative vicious transactional processes (Sutherland & Oswald, 2005; Spilt, Koomen, Thijs, & van der Leij, 2012; Thijs & Koomen, 2009).

The significant effect of the negative relationship condition on teachers’ tolerance of misbehavior supported the automatic impact of teachers’ mental representations of relationships with individual students because this effect was only found in the negative relationship condition (congruent with what we expected) compared to both control conditions. In contrast with the previous experiment, here we also found a difference between different known students based on relationship valence (negative relationship condition versus distant relationship condition) and not only a difference between known and unknown students. From a practical perspective, this result suggests that when in interactions with a particular student a negative mental relationship presentation is activated, the teacher will be less tolerant of subsequent student misbehavior impairing the teacher to identify as well as to respond sensitively to the actual needs of the students (Chang & Davies, 2009; Hamre & Pianta, 2001; Pianta, 1999).

No differences were found between the positive relationship condition and the control conditions. This may indicate that teachers do not always develop mental representations of positive dyadic relationships or that the activated positive mental representations of teachers may have less or no impact on the social information processing of the teacher. As seen in other cognition research, positive evaluations might be the default which can explain that the positive activated valence might be less interfering with the social information processing of the teacher (Fiske, 1981 in Banse, 2001). The difference in impact of teachers’ mental representations of negative and positive relationships is compatible with other research indicating that not positive but negative affect is significantly associated with teachers’ behavioral interactions with individual students (Roorda, Koomen, & Oort, 2012; Stulhman, & Pianta, 2002). Perhaps, in comparison with a positive valence, the negative valence was more intense and harder to regulate or inhibit, impacting the social information processing of the teachers (Nezlek & Kuppens, 2008).

No significant priming effects were found on teachers’ attributions of student control, preferred intervention strategies, or relational investment. Perhaps, the significant results may not have been detected because the effect of the prime did not last beyond the first question. Wentura and Rothermund (2014) discussed that effects in priming experiments only last in a time range from fractions of a second to maximally a few seconds. After reading the vignette and answering the first question, it is possible that the effect of the prime had already decayed before answering the other three questions. Future research (e.g., priming before each question) is needed to improve this experiment.

### Toward Understanding Teacher-Student Interactions

The results of the current study provide mixed support for the notion that teachers’ mental representations of dyadic relationships with individual students impact teachers’ affective and cognitive information processing. The results of the first experiment indicated that priming with familiar students delayed teachers’ affective information processing. However, no support for the valence-congruent impact of teachers’ mental representations was found. The second experiment supported the idea that teachers’ mental representations of relationships with individual students impact teachers’ cognitive information processing, but only in negative, conflictual relationships with students (and only concerning teachers’ tolerance of student problem behavior). Thus, this study partially confirmed our hypothesis that teachers’ mental representations of dyadic relationships impact teachers’ affective and cognitive information processing. Subsequently, more research is needed to pursue the investigation of the development and impact of teachers’ mental representations of dyadic relationships.

The current study also contributed to research on teacher-student relationships being one of the first to apply the priming method to teacher-student relationships. Priming research on these interpersonal relationships may be an important contribution to the existent literature that typically relies on teacher-report questionnaires of teacher-student relationships (Thijs et al., 2008). This innovative experimental approach may help to elucidate the affective and cognitive social information processing as underlying mechanisms in (transactional) teacher-student interactions (Dykas & Cassidy, 2011; Wentura & Rothermund, 2014). Despite the inconclusive results, the current study provided a step forward in the rising research on teacher-student relationships, both theoretically and methodologically. Future research can be inspired by the design and the results as well as the hurdles and suggestions in this study to move the field forward to a more comprehensive understanding of teacher-student relationships.

### Limitations and Future Research

In the discussion of our two experiments, several concerns have to be mentioned. Some limitations and suggestions for future research were already discussed above, concerning the use of neutral or ambiguous targets (first experiment) and the use of (more) primes (second experiment).

Consistent with findings on gender differences in teacher-student relationship quality (e.g., Spilt, Koomen, & Jak, 2012), more girls were selected in the positive relationship condition and more boys in the negative relationship condition. These relationship differences may be explained by the fact that boys tend to have poorer self-regulation skills, are less engaged, and more disruptive in the classroom than girls which seems to make it more difficult for teachers to form close and non-conflictual relationships with boys (Spilt, Koomen, & Jak, 2012; Weyns, Colpin, De Laet, Engels, & Verschueren, 2018). In our priming experiment, this led to unequal distributions of boys and girls across conditions. However, the authors did not know of any empirical data suggesting that gender may be a confound variable in affective priming experiments (cf., Herman et al., 2003).^5^

It would be interesting to investigate differences *between* teachers in developing mental representations of dyadic relationships. We can expect, for example, that teachers’ own attachment history will impact the development of teachers’ mental representations. Dismissive-avoidant teachers may devalue relationships to protect themselves from feeling vulnerable (because of discomfort with closeness) and may be less inclined to internalize experiences with students into mental representations. In contrast, anxious-preoccupied teachers who may easily worry about being worthy of love (fear of rejection) may be more readily to internalize negative experiences with a student and may thus more impeded by negative associations in mental representations of dyadic relationships in daily interactions with students (Bartholomew & Horowitz, 1991; Morris-Rothschild & Brassard, 2006; Spilt et al., 2011).^6^

## Conclusion

Using affective priming, our results provided mixed support for the idea that teachers’ mental representations influences teachers’ affective and cognitive social information processing. Priming with photographs of familiar students delayed teachers’ affective social information processing, irrespective of the positive, negative or neutral valence of the teacher-student relationship. Concerning teachers’ cognitive social information processing, teachers’ mental representations of negative relationships seemed to impact teachers’ tolerance: teachers’ were less tolerant of student misbehavior when primed by a photograph of a student with whom they had a negative relationship. Follow-up research may profit from the discussion of the strengths and the limitations of our experiments in order to move the field forward toward more advanced understanding of teacher-student relationships.

## Additional Files

The additional files for this article can be found as follows:

10.5334/pb.471.s1 Appendix 1. Table A1 Vignettes of Experiment 2 (in Dutch).

10.5334/pb.471.s2 Appendix 2. Results of the First Experiment Based on Log Reaction Times.

10.5334/pb.471.s3 Appendix 3. Results of the Experiments, 9 suspicious teachers excluded.

## Footnotes

This teacher got one picture of the Positive and one of the Neutral condition extra, to have the same amount of pictures in the experiments.

Distributions of Closeness and Conflict are always skewed (based on normative data: closeness and conflict are differently distributed, right versus left skewed). Accordingly, very few students who were very high on conflict (>15% highest) and at the same time very low on closeness (<25% lowest) were expected. In order to have enough students in the ‘negative relationship condition,’ our criterion resulting in a cut-off score on closeness <85%. Thus, having a lower score on conflict was decisive, but by adding the <85% criterion, students who were above-average on closeness were excluded. With respect to the ‘positive relationship condition’, based on the normative data, we could be confident to find students scoring high on closeness (>85%) and low on conflict (<25%).

Independent assessors (*n* = 29) rated the attractiveness of the photographs of the selected students used in the priming procedures on a scale form one to five. In this way, we wanted to control for facial attractiveness and exclude attractiveness as an alternative explanation for the priming effects because attractive students may be perceived as more likeable than less attractive students (based on Nummenmaa et al., 2008). To check for this, mean attractiveness ratings were computed for the neutral expression of each student and were subjected to a one-way independent sample ANOVA. Almost all the multiple comparisons of the ANOVA (at the .05 significance level using the Benjamini–Hochberg procedure for multiple comparison correction) were not significant referring to no significant differences in facial attractiveness across conditions. However, one of the multi comparison was significant: the students in the closeness relationship condition were significantly more attractive than the students in the distant relationship condition.

In priming experiments, reaction times are often positively skewed as is for our experiment. To reduce the skewness of our data, we conducted the same analyses using the natural log of teachers’ reaction time based on Field and Wright (2011). The conclusions based on these results remained the same (see Appendix 2).

To check this assumption, a post-hoc analysis was conducted on the raw data (after exclusion of block 1, outliers and errors) of the neutral primes of all the teachers (*n* = 1768): a simple independent sample test indicated no significant difference between boys (*M* = 638.49; *SD* = 213.36) and girls (*M* = 642.72; *SD* = 215.11) in reaction times (*t* = –.42, *p* > .05).

In a preliminary analysis in the current study, we did check the hypothesis that the attachment type of the teacher may moderate the impact of teachers’ mental representations of dyadic relationships on the outcomes. However, no significant moderating effects of dismissive or preoccupied attachment (measured by the Adult Attachment Questionnaire, Bartholomew & Horowitz, 1991) were found (probably due to power issues related to a small sample size because the study was not designed to detect between-subject differences).
