# Supplementary material for: Toward Empirical Evidence for Teachers’ Mental Representations of Dyadic Relationships With Students: Two Priming Experiments
Source: Psychol Belg. 2019 May 9;59(1):156–76. doi: 10.5334/pb.471 (PMC6625555; doi:10.5334/pb.471)
Supplement: Appendix 1. — Table A1 Vignettes of Experiment 2 (in Dutch). [file pb-59-1-471-s1.pdf]

APPENDIX 1

Table A1

Vignettes of Experiment 2 (in Dutch)

	Vignetten
1.	Je hebt net nieuwe leerstof uitgelegd aan de klas en een bijhorend werkblaadje hierover uitgedeeld. Je kijkt over de klas en ziet dat deze leerling aan het praten is met een buur.
2.	De leerlingen moeten een opstel schrijven. Een leerling is aan het treuzelen in plaats van te beginnen met schrijven.
3.	De kinderen zijn aan het knutselen. Ineens klinkt er een verontwaardigde uitroep van één van de kinderen door de klas. Wanneer je opkijkt zie je dat deze leerling het kunstwerkje van een andere leerling bij het voorbij lopen op de grond heeft laten vallen.
4.	Drie kinderen zijn mens-erger-je-niet aan het spelen, een dobbelspel waar vier spelers via een weg naar het midden van het bord moeten geraken. Een andere leerling wil graag meedoen en vraagt dit. Deze leerling, één van de spelers van het spel, reageert dat dit niet kan.
5.	De tijd die gepland is om aan muzische vorming te besteden, is bijna ten einde. Je maant aan om op te ruimen. Een leerling reageert hierop dat ze nog niet klaar is.
6.	De klas staat op het punt om met een toets te starten. De klas is stil. Op het moment dat je uitleg wil gaan geven, opent een leerling de lade van de lessenaar. Het schrift en enkele losse papieren van deze leerling glijden van de tafel. Deze leerling raapt ze langzaam één voor één op.
7.	Je bent reeds een uur met een groepsdiscussie bezig in de klas. Je stelt één van je leerlingen een vraag over het onderwerp van de groepsdiscussie, maar deze leerling lijkt afgeleid en hoort je niet.
8.	Vandaag ben je de vorderingen in de leerstof aan het nagaan. Wanneer je een vraag stelt aan een van je leerlingen, kijkt deze leerling jou fronsend aan.
9.	Deze leerling van je klas heeft juist verteld dat één van de andere jongens van de klas ‘lelijke woorden’ heeft gebruikt tijdens de speeltijd.
10.	De leerlingen hebben twintig minuten de tijd gekregen om hun rekentaak zelfstandig te maken. Eén van je leerlingen brengt jou de taak en meent klaar te zijn, maar je ziet dat verschillende vragen opengelaten zijn.
11.	Je bent de klas aan het verdelen in groepjes om aan een project te werken. Je hoort een leerling een opmerking geven over een klasgenoot die bij hem in de groep wordt ingedeeld.
12.	Een leerling in jouw klas heeft net ontdekt dat één van de schroeven waarmee de rug van de stoel is vastgemaakt, los zit. Deze leerling probeert de schroef terug vast te zetten door hieraan te duwen en trekken. Door het lawaai dat wordt gemaakt, raakt de klas afgeleid.
13.	Je hebt net een taak uitgelegd en de instructies gegeven voor de hele klas. De leerlingen beginnen vlijtig aan de taak. Deze leerling is aan het rondkijken en aan het mompelen in plaats van aan de taak te beginnen.

14.	De klas heeft net instructies gekregen om snel in een rij te gaan staan. De leerlingen schikken zich, met uitzondering van deze leerling. Deze leerling blijft zitten, werkend aan een tekening.
15.	Je deelt een werkblaadje over verkeer uit in de klas en alle leerlingen beginnen eraan te werken. Na enkele minuten merkt je op dat deze leerling uit het raam aan het kijken is en helemaal niet meer met het werkblaadje bezig is.
16.	Een leerling kan zijn knutselwerkje niet vinden en vraagt jou om te helpen. Deze leerling geraakt helemaal van streek.
17.	Je hebt juist een nieuwe les gegeven en hebt hierover werk uitgedeeld. Je kijkt door de klas en ziet dat deze leerling met een geo-driehoek kunstjes aan het doen is. Wanneer je deze leerling vraagt of er iets scheelt, zegt deze aan jou ‘ Ik kan het niet, het is te moeilijk voor mij’.
18.	De middagpauze is juist voorbij. Je bent de kinderen in de rij op de speelplaats gaan halen en jullie zijn samen naar de klas gelopen. Wanneer iedereen zit, start je met je les. Eén van je leerlingen komt tien minuten te laat binnen in de klas en gaat aan zijn tafel zitten.
19.	De leerlingen zijn in de wiskundeles zelfstandig aan het werk. Plots neemt deze leerling de rekenblokjes/rekenmachine van de buurman af.
20.	De afwerking van het moederdagcadeau is net afgerond in de klas. Deze leerling verwisselde het eigen kunstwerkje met een ander en had het reeds in de boekentas opgeborgen. De andere leerling van wie het kunstwerkje was, geraakt hierdoor van streek.
21.	Bij het onderwerp over de oorlog tijdens wereldoriëntatie, roept deze leerling plots luid en gepassioneerd wat hij er allemaal over weet doorheen heel de klas.
22.	Iedereen moest zeker zijn taak meebrengen op de laatste dag van de week. Dit was al vorige week gezegd en in de agenda geschreven. Deze leerling heeft zijn taak echter niet mee vandaag.
23.	Een leerling in jouw klas trapt de brooddoos van een klasgenootje kapot en loopt snel verder.
24.	Je ziet een leerling van jouw klas een duw geven aan een andere leerling. De leerling is normaal niet gewelddadig. De kans is groot dat dit gedrag van deze leerling uitgelokt werd door de pester van de klas.
	PRACTICE TRIALS
25.	Tijdens een bezinning wordt er een verhaaltje voorgelezen. Deze leerling lijkt aandachtig te kijken en te knikken als je de discussie over het verhaal aan het leiden bent. Verheugd om deze leerling aandachtig te zien, stel je deze leerling een vraag. Wanneer je de naam van deze leerling herhaalt, lijkt de leerling eerder geschrokken en kan deze leerling niet op jouw vraag antwoorden.
26.	Je bent bezig met een nieuwe regel over spelling. Je geeft een voorbeeld en vraagt hoe die regel toegepast kan worden op jouw voorbeeld. Een leerling steekt zijn hand op en je geeft aan dat hij mag antwoorden. Hij antwoordt dat zijn oma vandaag jarig is.
27.	Je hebt net de les uitgelegd en je hoort deze leerling zuchten.
28.	Je geeft een herhalingsles wereldoriëntatie. Alle studenten beantwoorden jouw vragen met gemak, maar als je deze leerling een vraag stelt lijkt deze helemaal verloren.
