# Supplementary material for: Toward Empirical Evidence for Teachers’ Mental Representations of Dyadic Relationships With Students: Two Priming Experiments
Source: Psychol Belg. 2019 May 9;59(1):156–76. doi: 10.5334/pb.471 (PMC6625555; doi:10.5334/pb.471)
Supplement: Appendix 2. — Results of the First Experiment Based on Log Reaction Times. [file pb-59-1-471-s2.pdf]

APPENDIX 2

Results of the First Experiment Based on Log Reaction Times

Before the start of the analysis of the first experiment, the first block (first 32 prime-targets stimuli) was removed because pre-exposure to the stimuli is recommended to investigate affective priming effects (Calvo & Nummenma, 2007). After the removal of outliers (> |3 *SD*|; 1.60%) and errors (not identifying the correct emotional expression; 2.12%), the Reaction time of the teachers was transformed to the natural log of their reaction times to reduce skewness (cf., Field & Wright, 2011). Subsequently, for each teacher, the average Log Reaction time for each condition was calculated. Teachers' Log Reaction time ranged from 5.04 to 7.90 ($M = 6.44$; $SD = .29$). Two repeated measures analyses of variance (ANOVA) on Log Reaction time were conducted in a 2 (Target: Happy vs. Angry) x 3 (Condition: Positive vs. Negative vs. Control) design. The first analysis included Distant relationship condition as control condition and the second analysis included the Unknown condition as control condition.

Concerning the analysis including the Distant relationship control condition, the results showed a significant main effect of Target ($F(1,52) = 7.69$, $p = .01$), indicating overall slower responses for Angry targets in comparison to Happy targets. The results showed no significant main effect of the within-subject factor Condition on Log Reaction time ($F(2,104) = 0.94$, $p = .40$). In addition, no interaction-effect between Condition and Target was found ($F(1.70,88.27) = 1.48$, $p = .24$ – Greenhouse-Geisser correction due to violation of sphericity with $\varepsilon = .85$), indicating no congruency effects (i.e., the effect of condition was the same across targets). Additionally, within-subject contrasts in the repeated measure ANOVA were conducted to compare the Positive relationship condition and Negative relationship condition with the Distant relationship control condition (see Table A1). No significant within-subject contrasts for the

Positive relationship condition ($F(1,52) = 0.54$, $p = .47$) and the Negative relationship condition ($F(1,52) = 2.07$, $p = .16$) compared to the Distant relationship control condition were found.

Concerning the analysis including the Unknown control condition, the results showed a significant main effect of Target ($F(1,53) = 12.29$, $p < .01$), indicating overall slower responses for Angry targets in comparison to Happy targets. The results showed also a significant main effect of the within-subject factor Condition on Log Reaction time ($F_{Unknown}(2,106) = 8.49$, $p < .01$). No interaction-effect between Condition and Target was found ($F(2,106) = 1.87$, $p = .16$), indicating no congruency effects (i.e., the effect of condition was the same across targets). Additionally, within-subject contrasts in the repeated measure ANOVA were conducted (see Table A1). Significant within-subject contrasts for the Positive relationship condition ($F(1,53) = 8.78$, $p = .01$) and the Negative relationship condition ($F(1,53) = 14.73$, $p < .01$) compared to the Unknown control condition were found. Teachers were slower in recognizing the emotional expressions in the Positive and Negative relationship conditions compared to the Unknown control condition.

Table A2

Descriptive Statistics of Teachers' Responses per Condition and Contrasts of the Repeated Measure ANOVA for Experiment 1

	**Condition (valence of prime)**					
	Positive relationship (P)	Negative relationship (N)	Control		Contrast compared to Control	
			Distant (D)	Unknown (U)	Distant	Unknown
**Target**	*M*(*SD*)	*M*(*SD*)	*M*(*SD*)	*M*(*SD*)		
Log Reaction time Happy	6.43(0.22)	6.42(0.21)	6.42(0.22)	6.39(0.20)		
Angry	6.46(0.23)	6.47(0.25)	6.46(0.25)	6.45(0.23)		
Total	6.45(0.23)	6.45(0.23)	6.44(0.24)	6.42(0.22)	P=D	**P>U***
					N=D	**N>U***

Note. * *p* <.05; All the within-subject contrasts were controlled for familywise error rate due to multiple comparisons using the Benjamini–Hochberg procedure and were still significant at the significance level of .05; Positive relationship condition = high on Closeness, low on Conflict; Negative relationship condition = low on Closeness, high on Conflict; Distant relationship control condition = low on Closeness, low on Conflict; Unknown control condition= unknown student.
