# Supplementary material for: Toward Empirical Evidence for Teachers’ Mental Representations of Dyadic Relationships With Students: Two Priming Experiments
Source: Psychol Belg. 2019 May 9;59(1):156–76. doi: 10.5334/pb.471 (PMC6625555; doi:10.5334/pb.471)
Supplement: Appendix 3. — Results of the Experiments, 9 suspicious teachers excluded. [file pb-59-1-471-s3.pdf]

APPENDIX 3

Results of the First Experiment, 9 suspicious teachers excluded

Concerning the analysis including the Distant relationship control condition, the results showed a significant main effect of Target ($F(1,43) = 5.56$, $p = .02$), indicating overall slower responses for Angry targets in comparison to Happy targets. The results showed no significant main effect of the within-subject factor Condition on Reaction time ($F(2,86) = 0.39$, $p = .68$). In addition, no interaction-effect between Condition and Target was found ($F(1.50,64.45) = 3.10$, $p = .07$ – Greenhouse-Geisser correction due to violation of sphericity with $\varepsilon = .75$), indicating no congruency effects (i.e., the effect of condition was the same across targets). Additionally, within-subject contrasts in the repeated measure ANOVA were conducted to compare the Positive relationship condition and Negative relationship condition with the Distant relationship control condition (see Table A1). No significant within-subject contrasts for the Positive relationship condition ($F(1,43) = 0.06$, $p = .80$) and the Negative relationship condition ($F(1,43) = 0.74$, $p = .40$) compared to the Distant relationship control condition were found.

Concerning the analysis including the Unknown control condition, the results showed a significant main effect of Target ($F(1,44) = 7.71$, $p =.01$), indicating overall slower responses for Angry targets in comparison to Happy targets. The results showed also a significant main effect of the within-subject factor Condition on Reaction time ($F(2,88) = 8.00$, $p <.01$). No interaction-effect between Condition and Target was found ($F(2,88) = 2.50$, $p =.09$), indicating no congruency effects (i.e., the effect of condition was the same across targets). Additionally, within-subject contrasts in the repeated measure ANOVA were conducted (see Table A1). Significant within-subject contrasts for the Positive relationship condition ($F(1,44) = 8.77$, $p = .01$) and the Negative relationship condition ($F(1,44) = 12.11$, $p <.01$) compared to the Unknown

control condition were found. Teachers were slower in recognizing the emotional expressions in the Positive and Negative relationship conditions compared to the Unknown control condition.

Results of the Second Experiment, 9 suspicious teachers excluded

Table A1 also depicts the descriptive statistics of teachers' responses on the four questions in the vignette task: Tolerance of student's behavior, Attribution of low control, Limits setting (vs. support), and Relational investment. Concerning the analysis including the Distant relationship control condition, the within-subject contrasts of the Positive and Negative relationship condition compared to the Distant relationship control condition revealed a significant effect for the Negative relationship condition ($F(1,42) = 4.79$, $p = .03$), but not for the Positive relationship condition ($F(1,42) = 0.73$, $p = .40$) for the first question about Tolerance of student's behavior. Accordingly, teachers' responses were significantly lower on tolerance in the Negative relationship condition in comparison to Distant relationship control condition. Regarding the second question about Attribution of low control, no significant within-subject contrasts of the Positive ($F(1,42) = 0.01$, $p = .95$) and Negative ($F(1,42) = 0.07$, $p = .79$) relationship conditions compared to the Distant relationship control condition were found. Also for the third question about Limits setting (vs. support), no significant within-subject contrasts of the Positive ($F(1,42) = 0.49$, $p = .49$) and Negative ($F(1,42) = 0.01$, $p = .92$) relationship conditions compared to the Distant relationship control condition were found. Finally, for the fourth question about Relational investment, no significant within-subject contrasts of the Positive ($F(1,42) = 3.44$, $p = .07$) and Negative ($F(1,42) = 2.22$, $p = .14$) relationship conditions compared to the Distant relationship control condition were found.

Concerning the analysis including the Unknown control condition, the within-subject contrasts of the Positive and Negative relationship condition compared to the Unknown control

condition revealed a significant effect for the Negative relationship condition ($F(1,43) = 5.48$, $p = .02$), but not for the Positive relationship condition ($F(1,43) = 0.86$, $p = .36$) for Tolerance of student's behavior. As seen in the comparison with the Distant relationship control condition, teachers' responses were significantly lower on tolerance in the Negative relationship condition in comparison to the Unknown control condition. Regarding the second question about Attribution of low control, no significant within-subject contrasts of the Positive ($F(1,43) = 0.04$, $p = .85$) and Negative ($F(1,43) = 0.05$, $p = .83$) relationship conditions compared to the Unknown control condition were found. Also for the third question about Limits setting (vs. support), no significant within-subject contrasts of the Positive ($F(1,43) = 0.04$, $p = .85$) and Negative ($F(1,43) = 0.69$, $p = .41$) relationship conditions compared to the Unknown control condition were found. Finally, for the fourth question about Relational investment, no significant within-subject contrasts of the Positive ($F(1,43) = 0.14$, $p = .71$) and Negative ($F(1,43) = 0.04$, $p = .85$) relationship conditions compared to the Unknown control condition were found.

Table A3

Descriptive Statistics of Teachers' Responses per Condition and Contrasts of the Repeated Measure ANOVA

	**Condition (valence of prime)**					
	Positive relationship (P)	Negative relationship (N)	Control		Contrast compared to Control	
			Distant (D)	Unknown (U)	Distant	Unknown
**Target**	*M*(*SD*)	*M*(*SD*)	*M*(*SD*)	*M*(*SD*)		
	**EXPERIMENT 1**					
Reaction time						
Happy	646.56(150.31)	633.42(140.86)	639.04(152.18)	618.12(134.41)		
Angry	667.40(169.02)	685.97(194.17)	672.72(188.62)	660.24(170.11)		
Total	656.98(159.67)	659.70(167.52)	655.88 (170.40)	639.18(152.26)	P=D	**P>U** **
					N=D	**N>U** **
	**EXPERIMENT 2**					
Response						
Tolerance of behavior	4.66(1.47)	4.46(1.16)	4.84(1.22)	4.86(1.22)	P=D	P=U
					**N<D***	**N<U***
Attribution of low control	4.72(1.14)	4.72(1.18)	4.66(1.35)	4.76(1.10)	P=D	P=U
					N=D	N=U
Limits setting (vs. support)	5.59(1.38)	5.75(1.34)	5.74(1.50)	5.55(1.32)	P=D	P=U
					N=D	N=U
Relational investment	6.59(1.63)	6.69(1.41)	6.82(1.52)	6.65(1.67)	P=D	P=U
					N=D	N=U

Note. * p <.05; All the within-subject contrasts were controlled for familywise error rate due to multiple comparisons using the Benjamini–Hochberg procedure and those with ** were still significant at the significance level of .05 (cf., Benjamini & Hochberg, 1995); Positive relationship condition = high on Closeness, low on Conflict; Negative relationship condition = low on Closeness, high on Conflict; Distant relationship control condition = low on Closeness, low on Conflict; Unknown control condition= unknown student
